# Natural Language Processing for Surveillance of Cervical and Anal Cancer and Precancer: Algorithm Development and Split-Validation Study

**DOI:** 10.2196/20826

**Published:** 2020-11-03

**Authors:** Carlos R Oliveira, Patrick Niccolai, Anette Michelle Ortiz, Sangini S Sheth, Eugene D Shapiro, Linda M Niccolai, Cynthia A Brandt

**Affiliations:** 1 Department of Pediatrics Yale University School of Medicine New Haven, CT United States; 2 Department of Obstetrics, Gynecology, and Reproductive Sciences Yale University School of Medicine New Haven, CT United States; 3 Departments of Epidemiology of Microbial Diseases Yale School of Public Health New Haven, CT United States; 4 Departments of Emergency Medicine, Biostatistics, and Health Informatics Yale Schools of Medicine and Public Health New Haven, CT United States; 5 Veteran Affairs Connecticut Healthcare System West Haven, CT United States

**Keywords:** natural language processing, automated data extraction, human papillomavirus, surveillance, pathology reporting, cervical cancer, anal cancer, precancer, cancer, HPV, accuracy

## Abstract

**Background:**

Accurate identification of new diagnoses of human papillomavirus–associated cancers and precancers is an important step toward the development of strategies that optimize the use of human papillomavirus vaccines. The diagnosis of human papillomavirus cancers hinges on a histopathologic report, which is typically stored in electronic medical records as free-form, or unstructured, narrative text. Previous efforts to perform surveillance for human papillomavirus cancers have relied on the manual review of pathology reports to extract diagnostic information, a process that is both labor- and resource-intensive. Natural language processing can be used to automate the structuring and extraction of clinical data from unstructured narrative text in medical records and may provide a practical and effective method for identifying patients with vaccine-preventable human papillomavirus disease for surveillance and research.

**Objective:**

This study's objective was to develop and assess the accuracy of a natural language processing algorithm for the identification of individuals with cancer or precancer of the cervix and anus.

**Methods:**

A pipeline-based natural language processing algorithm was developed, which incorporated machine learning and rule-based methods to extract diagnostic elements from the narrative pathology reports. To test the algorithm’s classification accuracy, we used a split-validation study design. Full-length cervical and anal pathology reports were randomly selected from 4 clinical pathology laboratories. Two study team members, blinded to the classifications produced by the natural language processing algorithm, manually and independently reviewed all reports and classified them at the document level according to 2 domains (diagnosis and human papillomavirus testing results). Using the manual review as the gold standard, the algorithm’s performance was evaluated using standard measurements of accuracy, recall, precision, and F-measure.

**Results:**

The natural language processing algorithm’s performance was validated on 949 pathology reports. The algorithm demonstrated accurate identification of abnormal cytology, histology, and positive human papillomavirus tests with accuracies greater than 0.91. Precision was lowest for anal histology reports (0.87, 95% CI 0.59-0.98) and highest for cervical cytology (0.98, 95% CI 0.95-0.99). The natural language processing algorithm missed 2 out of the 15 abnormal anal histology reports, which led to a relatively low recall (0.68, 95% CI 0.43-0.87).

**Conclusions:**

This study outlines the development and validation of a freely available and easily implementable natural language processing algorithm that can automate the extraction and classification of clinical data from cervical and anal cytology and histology.

## Introduction

Precision public health is a rapidly evolving field that focuses on promoting the health of a population through the application of technology [1]. A key priority in precision public health is the development of new informatics approaches to optimize the use of vaccines for the prevention of disease. Some of the more successful vaccine informatics applications postlicensure include using text-mining techniques to automate the tracking of adverse immunization outcomes and the use of emergency department notes as an early warning sign for outbreaks of vaccine-preventable diseases. Automation of biosurveillance and timely identification of infectious diseases is of particular importance to public health, as it allows for better planning and distribution of limited resources [2-4].

Persistent infection with human papillomavirus (HPV) can result in precancerous anogenital lesions as well as invasive cancer. In the United States, approximately 25,000 cases of anogenital cancers are diagnosed every year, with cervical and anal cancer being the majority (75%) of these [5]. Over 90% of these cases are attributable to infection with HPV types that are preventable by the use of recommended HPV vaccines [5-7]. Although HPV vaccines have high proven efficacy, the way we use these vaccines to prevent HPV cancers is still in need of improvement [8]. Accurate identification and tracking of new cases of HPV cancers is an important step toward the development of strategies that optimize the use of HPV vaccines.

Surveillance for HPV-associated outcomes is critical for monitoring the progress of immunization programs and identifying targets for improvement. Surveillance for HPV cancers, however, has been a formidable challenge. Most of the clinical data needed to diagnose a patient with an HPV-related cancer, or precancer, are stored in pathology reports. Normally, pathology reports are stored in a narrative format and contain several lines of text that can include nondiagnostic information, such as medical history or clinical indications for screening [9]. Although a manual review of these free-text pathology reports is the most accurate case-finding method, it is a laborious process that can become too impractical for large-scale surveillance projects. To facilitate data capture and analysis, considerable efforts have been made to promote processes that encourage pathologists to document their findings in a specific format and using standardized terminology [10]. However, most efforts to incorporate standardized reporting have yet to be consistently implemented by health care providers and institutions [11].

To develop an accurate and scalable surveillance platform for HPV vaccine-preventable cancers, it is critical to first overcome the challenge of narrative data-abstraction. A potential solution to this data-abstraction problem is automation with computational tools, such as natural language processing (NLP). NLP is an increasingly used approach that combines informatics and linguistic techniques to automatically identify and extract key concepts or phrases embedded in a narrative text [12]. Although NLP has been successfully applied for the surveillance of several cancers (eg, colon, hepatic, and bladder cancer), it has been underutilized for the surveillance of HPV cancers and precancers [12-15].

As a first step toward achieving automated surveillance of HPV vaccine-preventable diseases, we developed an NLP algorithm aiming to extract information from cervical and anal pathology reports and classify these reports based on the pathologist’s final diagnosis. The objective of this study was to assess the accuracy of our NLP algorithm for the identification of individuals with cancer or precancer of the cervix and anus.

## Methods

### Study Design and Setting

This study used data generated from the HPV Vaccine Effectiveness Project, a large-scale population-based study aiming to determine the effectiveness of the HPV vaccine [16]. In support of this ongoing project, an NLP algorithm was developed to convert narrative pathology reports into structured data that can be queried to identify individuals who had HPV-related abnormalities in their cervical or anal pathology report. To build and evaluate this NLP algorithm, a split-validation method was used, wherein 2 sets of full-length cervical and anal pathology reports were randomly selected from 4 different clinical pathology laboratories within the Yale–New Haven Health System participating in the HPV Vaccine Effectiveness Project. The first set of reports was used to build the algorithm (ie, the training set, n=100), and the second set was used for testing the accuracy of the algorithm (ie, the validation set, n=1000). Pathology reports were extracted between January 1, 2010 and December 31, 2018 and deidentified for both the development and testing phases of this study.

### NLP Algorithm Development

We developed a pipeline-based NLP algorithm that incorporated both machine learning and rule-based methods to extract and classify diagnostic elements (histopathology, cytopathology, and HPV test results) from narrative pathology reports. Various software platforms have been developed to automatically annotate and process clinical notes based on the Unstructured Information Management Architecture framework [17-19]. Our pipeline was built using CLAMP (Clinical Language Annotation, Modeling, and Processing) software, because it is open-source, modular, free-to-use, and specifically designed to process and analyze clinical text [20]. Our pipeline combined several existing and well-validated text processing components [21-27] and built on these components with newly developed HPV-specific ontologies and postprocessing features.

### NLP Data Extraction

The first steps of our pipeline involved using CLAMP’s existing algorithms to preprocess each report and apply a series of if-then rules to parse and enumerate each sentence and word within the full-length report (ie, a sentence detector and word tokenizer, respectively) [24]. Next, we used a supervised machine learning approach to assign each enumerated token (ie, each word or set of words) a tag based on its part of speech (eg, verb, noun, etc) [28]. A more in-depth description of the pipeline's individual preprocessing components can be found in Multimedia Appendix 1. We then implemented an existing named entity recognizer program to identify key concepts within the narrative text [29]. This named entity recognizer program utilizes a dictionary-based approach to match concepts in pathology reports to terms in a dictionary derived from the Unified Medical Language System Metathesaurus [27]. To more robustly account for variations in HPV-related concepts, we also constructed an HPV-cancer dictionary and incorporated it into the algorithm. This custom HPV-cancer dictionary leveraged over a decade of experience and expertise in HPV-cancer surveillance through collaboration with seasoned epidemiologists from HPV Vaccine Impact Monitoring Project Across Connecticut, a collaborative project between the Connecticut Emerging Infections Program at Yale School of Public Health; the Connecticut Department of Public Health; and the Centers for Disease Control and Prevention [30]. We have contributed our HPV dictionary (ie, ontology) to the National Center for Biomedical Ontology BioPortal platform [31], where it is openly available for other users to develop further.

### NLP Data Classification

After implementing the dictionary-based named entity recognizer, we applied newly developed heuristic rules to analyze and relabel each concept based on their context in the report. For example, a series of if-then rules were employed to identify different sections of the report (eg, clinical history, molecular diagnosis, primary diagnosis, etc) and determine when an HPV-related diagnosis was being stated in the report as a historical piece of information and when it was being stated in the context of the current specimen. Further details and examples of the key if-then rules are shown in Multimedia Appendix 1.

We also implemented an extensively validated rule-based negation algorithm [23] to allow us to differentiate when a recognized concept was being negated or stated with uncertainty based on the words that preceded or followed the identified concept (eg, “negative for abnormalities” or “abnormalities were not found”). Once all entities were named, coded, and contextualized, the algorithm generated a structured output (matrix) that was suitable for further processing. For the last step of the algorithm, the structured output was used to summarize and classify each report, at the document level, in 2 key domains: final diagnosis (using the Bethesda Classification system) and results of HPV tests (if performed). To enable the reproducibility of this study, our pipeline was freely available for research through CLAMP [32] and is archived [33]. To facilitate its application, we also provide a step-by-step video demonstration of this pipeline [33].

### Classification Validation

To test the algorithm’s classification accuracy, 2 study team members, blinded to the classifications produced by the NLP algorithm, manually and independently reviewed all pathology reports in the validation set and classified them at the document level according to the same 2 domains (diagnosis and HPV testing results). Disagreement among the 2 manual-review adjudicators was resolved by discussion with a third investigator.

**Figure 1:**
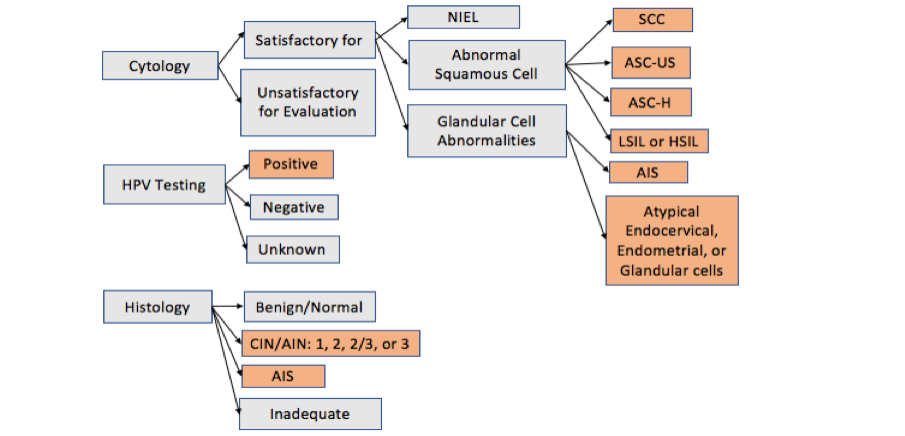
Diagrammatic representation of the classification process for pathology reports (colored indicates abnormal pathology). AIN: anal intraepithelial lesion; AIS: adenocarcinoma in situ; ASC-US: atypical squamous cells of undetermined significance; ASC-H: atypical squamous cells—cannot exclude high-grade squamous intraepithelial lesion; CIN: cervical intraepithelial lesion;HSIL: high-grade squamous intraepithelial lesion; LSIL: low-grade squamous intraepithelial lesion; NIEL: negative for intraepithelial lesion; SCC: squamous cell carcinoma.

For the primary analysis, we tested this algorithm’s accuracy for the identification of HPV-related pathology. The primary outcome—abnormal pathology—was grouped as a dichotomous variable and defined, for cytology reports, as a final diagnosis of atypical squamous cells or greater, and for histology reports, as intraepithelial neoplasia grades 2 or greater. A summary of the classification process for the primary outcome is shown in Figure 1.

### Statistical Analysis

The algorithm’s performance was evaluated using the manual review classifications as the standard. Accuracy, precision, recall, and F-measure were calculated as follows: *accuracy* = (*true positives* + *true negatives*) / (*true positives* + *true negatives* + *false positives* + *false negatives*); *precision* = *true positives* / (*true positives* + *false positives*); *recall* = *true positives* / (*true positives* + *false negatives*); *F-measure* = 2 × (*precision* × *recall*) / (*precision* + *recall*). Statistical analyses were conducted using Stata statistical software (version 15; StataCorp LLC). This protocol was approved by the institutional review board of Yale University (protocol number 2000024708).

## Results

Out of 1000 pathology reports originally selected for the validation set, 51 were excluded after manual review because they were (1) reports with misclassified specimens (ie, not anal or cervical tissue), (2) duplicate reports, or (3) incomplete reports. Testing of the NLP algorithm’s accuracy was performed on 949 pathology reports (anal cytology n=94; anal histology n=86; cervical cytology n=403; cervical histology n=366). HPV tests were documented on 303 reports (cervical n=265; anal cytology n=38), of which 121 (40%) had positive results for HPV. A summary of the highest-grade diagnosis based on manual review of the 949 pathology reports is shown in Table 1. Most of the biopsies performed revealed either normal or low-grade (362/452, 80%) lesions, and most of the cytologic specimens were negative for intraepithelial lesions (302/497, 61%).

**Table 1:** Summary of results from the manual review of the validation set.

Test	Cervical (n=769), n ( %)	Anal (n=180), n (%)	Total (N=949), n
Cytology	403 (81.1)	94 (18.9)	497
Negative for intraepithelial lesion	255 (84.4)	47 (15.6)	302
Atypical squamous cells of undetermined significance	44 (68.8)	20 (31.3)	64
Atypical squamous cells—cannot exclude high-grade squamous intraepithelial lesion	57 (98.3)	1 (1.7)	58
Low-grade squamous intraepithelial lesion	16 (84.2)	3 (15.8)	19
Glandular abnormality	14 (82.4)	3 (17.6)	17
Unsatisfactory specimen	17 (45.9)	20 (54.1)	37
HPV^a^ test performed	206 (85.8)	34 (14.2)	240
Positive	91 (84.3)	17 (15.7)	108
Histology	366 (81.0)	86 (19.0)	452
Benign	153 (77.3)	45 (22.7)	198
Squamous intraepithelial lesion grade 1	138 (84.1)	26 (15.9)	164
Squamous intraepithelial lesion grade 2+	75 (83.3)	15 (16.7)	90

^a^HPV: human papillomavirus.

For the primary analysis, the NLP algorithm accurately identified abnormal cytology, histology, and positive HPV tests with accuracies ≥0.91 in all specimens (Table 2). Precision was lowest for anal histology reports (0.87, 95% CI 0.59-0.98) and highest for cervical cytology (0.98, 95% CI 0.95-0.99). The NLP algorithm missed 2 out of the 15 abnormal anal histology reports, which led to relatively low recall (0.68, 95% CI 0.43-0.87).

**Table 2:** Performance of NLP algorithm on the validation set, N = 949.

Variable		Precision (95% CI)	Recall (95% CI)	F-measure (95% CI)	Accuracy (95% CI)
**Abnormal cytology^a^**					
	Cervical	0.98 (0.95-0.99)	1.00 (0.97-1.00)	0.99 (0.98-1.00)	0.99 (0.98-1.00)
	Anal	0.93 (0.76-0.99)	1.00 (0.86-1.00)	0.96 (0.91-1.00)	0.98 (0.93-0.99)
**HPV^b^ testing**					
	Positive	0.95 (0.89-0.98)	1.00 (0.97-1.00)	0.97 (0.95-0.99)	0.99 (0.98-1.00)
**Abnormal histology**					
	CIN^c^ grade 2+	0.89 (0.80-0.95)	0.93 (0.85-0.98)	0.91 (0.86-0.96)	0.96 (0.94-0.98)
	AIN^d^ grade 2+	0.87 (0.59-0.98)	0.68 (0.43-0.87)	0.76 (0.61-0.92)	0.91 (0.82-0.96)
**Average performance^e^**					
	Abnormal test	0.94 (0.91-0.97)	0.96 (0.92-0.98)	0.94 (0.93-0.97)	0.97 (0.96-0.98)

^a^Abnormalities include atypical squamous cells of undetermined significance, atypical squamous cells—cannot exclude high-grade squamous intraepithelial lesion, low-grade squamous intraepithelial lesion, and glandular cell abnormalities.

^b^HPV: human papillomavirus.

^c^CIN: cervical intraepithelial lesion.

^d^AIN: anal intraepithelial lesion.

^e^Includes results from both cytology and histology.

## Discussion

In this paper, we described the development and validation of an NLP instrument that can be used for both data extraction and classification of cytology and histology reports of the cervix and anus. Based on these initial data, our NLP algorithm can classify whether a cytology or histology specimen was abnormal and whether any HPV tests resulted positive, with an accuracy 91%. At the document level, this algorithm had an average recall (also known as sensitivity) of 96% and precision (also known as positive predictive value) of 94%. This demonstration of accuracy is an important first step toward the development of a tool that can facilitate the automation of surveillance for HPV vaccine-preventable cancers and precancers.

There is an increasing body of evidence showing the merits of an NLP system over manual review for data extraction and document classification for disease surveillance [34,35]. A key contribution of this study is the integration and application of well-validated NLP methodologies to solve a real-world public health problem. Most individual components included in our NLP pipeline have been previously validated. Using a commonly used corpus (SemEval-2014), Soysal et al [20] demonstrated that CLAMP’s named entity recognizer algorithm had superior precision to those of other commonly used platforms (CLAMP: 0.77; MetaMa*P*: 0.55; cTAKES: 0.46). In the same study [20], the performance accuracy of other key components (tokenizer, sentence boundary detector, part-of-speech tagger, and section detector) were evaluated using the MiPACQ clinical corpus and were also found to have a high accuracy (>92%). In our study, we did not aim to develop novel NLP strategies or components. However, one of the key strengths in our approach is that we were able to leverage the experience of HPV surveillance experts to assemble an extensive list of HPV-related terms to optimize named entity recognition.

This study has several other notable strengths. First, this study is among the first to evaluate the accuracy of an NLP algorithm to identify cases of HPV-related precancers. Although precancerous diagnoses are routinely made, these data are not systematically collected by most surveillance systems. These diagnoses, however, have public health significance as they can be used to monitor the impact of HPV vaccines. Our NLP algorithm provides an efficient way to use existing resources to measure the extent to which HPV vaccines reduce the burden of disease at the population level and identify areas to strengthen immunization programs. Automating the identification of precancers may also have clinical applications. For example, following an abnormal cytology result, a patient is usually kept under close surveillance for months. After an abnormal cytology screen, the appropriate management can vary from more frequent follow-up tests to immediate treatment with surgical excision. Automation of the detection of precancerous abnormalities in cytology or histology can be incorporated into clinical decision support tools to ensure patients are appropriately linked to care and are receiving timely follow-up.

An additional strength of this study is in the application of our NLP algorithm to accurately detect cases of anal cancer and precancer. To our knowledge, we are the first to provide a tool specifically designed for this purpose. Efforts to monitor the impact of HPV vaccination on oncogenic outcomes have focused mainly on cervical cancer and women. With the increased recognition that HPV also causes cancer in men and the increasing rate of these cancers in the young adult population [25,26], it is important to determine if the HPV vaccine's deployment can be optimized to reduce the burden of disease in both sexes. A surveillance system with these outcomes may be especially valuable to investigators and public health officials in assessing the impact of various immunization strategies in both males and females.

Additional improvements can optimize the performance of this algorithm for implementation in routine public health surveillance or clinical practice. For example, we only used reports from a single health care system (Yale New Haven Health), which likely limited the variability found in both the structure and language in the pathology reports. Thus, future work is needed to validate this tool's portability to other health care systems where pathology practices may differ. An additional area of improvement is in the preprocessing. After initial manual review of pathology reports, we had to exclude several reports that were incomplete or were misclassified in the electronic medical record. To be useful as a real-time surveillance tool, future iterations of this NLP algorithm will need to address the potential for misclassification at the onset. An additional limitation of this tool is that it was developed as a means to identify cases of cancer and precancer at the document level and not at the patient level. As many individuals have more than one pathology report in their record, to be useful as an automated surveillance method, more postprocessing will be needed to deal with duplicates or disparate findings at the patient level.

In this study, we detail the development of a freely available and easily implementable NLP algorithm that can automate the extraction of clinical data from cervical and anal cytology and histology reports. We show that with this algorithm, it is possible to accurately detect patients with HPV-related abnormalities at these anatomical sites. These data provide preliminary support for the use of our NLP instrument for the surveillance of HPV cancer and precancer of the cervix and anus.

## Appendix

Multimedia Appendix 1 Supplementary methods.

## Abbreviations

CLAMP: Clinical Language Annotation, Modeling, and Processing
HPV: human papillomavirus
NLP: natural language processing
