# Supplementary material for: Natural Language Processing for Surveillance of Cervical and Anal Cancer and Precancer: Algorithm Development and Split-Validation Study
Source: JMIR Med Inform. 2020 Nov 3;8(11):e20826. doi: 10.2196/20826 (PMC7671846; doi:10.2196/20826)
Supplement: Multimedia Appendix 1 [file medinform_v8i11e20826_app1.docx]

**Supplementary Methods**

Although there has been increasing interest in the application of NLP for public health and medical research, the adoption of NLP has been slow. This is partially due to the relative lack of reproducibility of the work and generalizability of the algorithms. A key strength in our approach is the use of an open-source platform (CLAMP) that is specifically tailored to clinical text. CLAMP is a stand-alone application that uses the UIMA framework. This platform can be installed on both Windows and Macintosh computers. The only prerequisite necessary to use this platform is JRE 1.8 (Java Runtime Environment). Below we provide a more detailed overview of the NLP features incorporated in our pipeline.

**Rule-based Text Annotation**

To fine-tune our pipeline's performance, we build a set of post-processing if-then rules using the Unstructured Information Management Architecture (UIMA) language and the Apache Ruta Rule Engine incorporated in CLAMP.[1] We added the rules to our NLP program to address issues that are specific to both pathology reports and our variables of interest (e.g., HPV testing, cytology/histology final diagnoses). These rules are used for modifying certain annotations based on their context relative to the other annotations. Rule elements consist of four parts: A matching condition, a quantifier, a list of conditions, and a list of actions. A few examples of these rules are detailed below:

1. HPV Test Rule: A "negative test" tag was made, which identified variations of terms denoting a negative test result (e.g., negative, not detected, below the threshold, non-reactive). Next, we used the Ruta Rule engine to specify that if a negative test tag was found before or after (up to 25 tokens) the mention of an HPV test, it needs to set the tag for the HPV test to "HPVNegative.” In turn, this instructs the summarizing program both that an HPV test was performed and that the result of that HPV test was in fact negative. Similarly, a "positive tag" was made and a rule was established so that when the positive tag was found before or after the mention of an HPV test, the Ruta Rule engine will switch the HPV test label to “HPVPositive." The following code is an example of this HPV test rule:

BLOCK(ForEach) Document{} {

ClampNameEntityUIMA{FEATURE("semanticTag", "Negative")}

BaseToken??{REGEXP(".{0,30}")}

ClampNameEntityUIMA{ FEATURE( "semanticTag", "HPV") -> GATHER(ClampRelationUIMA, "entFrom"=3, "entTo"=1), SETFEATURE("semanticTag","HPVNegative") }

The first line of code (“BLOCK(ForEach) Document”) tells the program to execute the containing rule in each one of the enclosed documents. The rule consists of four elements (shown above in red, yellow, blue and green). First, is the matching condition, which finds any token that CLAMP has annotated as “Negative” ({FEATURE("semanticTag", "Negative")}). The second element is the quantifier, which tells the program to match on a token that is 0-30 characters long (*BaseToken??{REGEXP(".{0,30}")}).* The third element is the list of conditions. In this case, the condition is set as the occurrence of a "Negative" annotation following the HPV test annotation (*FEATURE( "semanticTag", "HPV")*). Finally, the fourth step is the action that is to be taken if the condition is met (*SETFEATURE("semanticTag","HPVNegative")*)*.* This action tells the program that if it finds the annotation of an HPV test in the context of a negative annotation, it needs to change the annotation of “HPV” to “HPVNegative."

1. Historical Context Rule: For this rule, if a variation of the word "history" (e.g., previous or h/o) occurs before any mention of the patient's final diagnosis, the program changes the tag of that particular diagnosis to "History." This annotation, in turn, instructs the classification algorithm not consider that particular mention of a pathologic diagnosis as the report's final diagnosis. For example, if the report mentions the patient had a “history of negative for intraepithelial lesion (NIEL),” this would not be counted as the patient’s final current diagnosis. The following code is an example of this rule:

*BLOCK(ForEach) Document{} {*

*ClampNameEntityUIMA{ FEATURE("semanticTag", "History")}*

*BaseToken??{REGEXP(".{0,30}")}*

*ClampNameEntityUIMA{ FEATURE( "semanticTag", "NIEL") -> GATHER(ClampRelationUIMA, "entFrom"=3, "entTo"=1),*

*SETFEATURE("semanticTag","History") };*

Similar to the previous rule, this code consists of four elements. First, is the matching condition, which finds any token that CLAMP has annotated as “History” (*FEATURE(“semanticTag”, “History”)*). The second element is the quantifier, which tells the program to match on a token that is 0-30 characters long (*BaseToken??{REGEXP(".{0,30}")}).* The third element is the list of conditions; in this case, the condition is the proximity of the annotation "History" to the annotation of a NIEL diagnosis (*FEATURE( "semanticTag", "NIEL")*). Last is the action, which causes the program to change the annotation of "NIEL" to "History" if it occurs after an annotation of "History" (*SETFEATURE("semanticTag","History")*).

1. Anatomical Rule: The reports we examined often provided diagnoses from samples collected from numerous anatomical locations within a single report (i.e., cervix, vulva, vagina, or anus). To account for this, we added rules to extract only the locations we were interested in. For example, if the terms for vulva or vagina were listed in the report, they were tagged as “NotDiagnosis." Then, using a code similar to what is shown in the examples above, we instructed the program to exclude any diagnoses that were mentioned in reference to a vulvar or vaginal specimen.
2.
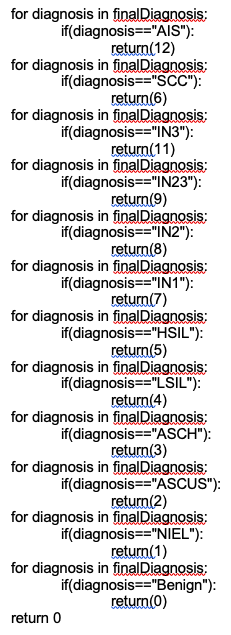
Multiple Grades Rule: Biopsy reports often contain multiple diagnoses for the same anatomical site depending on how many samples were collected during a procedure. For example, a single report for a patient may list the diagnosis of cervical intraepithelial neoplasia (CIN) grade 1 for a sample collected at the 9 o'clock position of the cervix and a diagnosis of CIN 3 for the sample collected at the 6 o'clock position of the cervix during the same procedure. To account for this, we incorporated a script (python) into the CLAMP pipeline to extract the annotations from CLAMP, enter them into a readable table, and identify the highest-grade lesion in the document. A sample of the script is provided on the right. This code works by looping through all the diagnoses in the report until it finds a match. In the first loop, if it finds a diagnosis of adenocarcinoma in situ (AIS), it will terminate the loop and return the number 12, which corresponds to the code for AIS (see Table S1 for other diagnosis codes). If it does not find "AIS," it will move on to the next line and look for squamous cell carcinoma (SCC), the second diagnosis on the list. If it does not find an SCC match, it will continue to loop through the output until it either finds a matching diagnosis or reaches the lowest option (CIN1/Benign).

| **Table S1. Extracted Diagnostic Entities by Specimen Type** | | | |
| --- | --- | --- | --- |
| **Specimen** | **Diagnosis** | **Numerical Output Code** | **Examples of Entities** |
| **Cytology (Pap)** | Benign | 0 | Benign |
|  |  |  | No significant abnormality |
|  | NIEL | 1 | Negative for intraepithelial lesions |
|  |  |  | Negative for dysplastic cells |
|  | ASC-US | 2 | Atypical squamous cells of undetermined significance |
|  |  |  | Abnormal squamous cells of undetermined significance |
|  | ASC-H | 3 | Atypical squamous cells cannot exclude a high-grade lesion |
|  |  |  | Atypical squamous cells of undetermined significance, cannot exclude a high-grade lesion |
|  | LSIL | 4 | Low-grade squamous intraepithelial lesion |
|  |  |  | Low-grade intraepithelial lesion |
|  | HSIL | 5 | High-grade squamous intraepithelial lesion |
|  |  |  | High-grade intraepithelial lesion |
|  | SCC | 6 | Squamous Cell Carcinoma |
|  |  |  | Squamous Carcinoma |
|  |  |  |  |
| **Histology (Biopsy)** | CIN1 or AIN1 | 7 | Intraepithelial lesion 1/I  Intraepithelial Lesion grade 1-3 |
|  |  |  | Mild dysplasia |
|  | CIN2 or AIN2 | 8 | Intraepithelial lesion 2/II |
|  |  |  | Moderate dysplasia of cervix |
|  | CIN or AIN 2/3 | 9 | Intraepithelial lesion 2/3 |
|  |  |  | Moderate to severe dysplasia |
|  | CIN 3 or AIN3 | 11 | Intraepithelial neoplasia 3/III |
|  |  |  | Severe dysplasia |
|  | AIS | 12 | Adenocarcinoma in situ |
| **All Reports** | Satisfactory | 13 | This specimen is satisfactory for evaluation |
|  | Unsatisfactory | 14 | Unsatisfactory due to insufficient cellularity |

**Sentence Detector**

The sentence detector component we used in the pipeline is the default sentence detector that is included in CLAMP.[2] It works by annotating the characters between two punctuation marks. Figure S1 is an example of the sentence detector being applied to a section of the pathology report.

**Figure S1. Sample biopsy annotated with the sentence detector.**


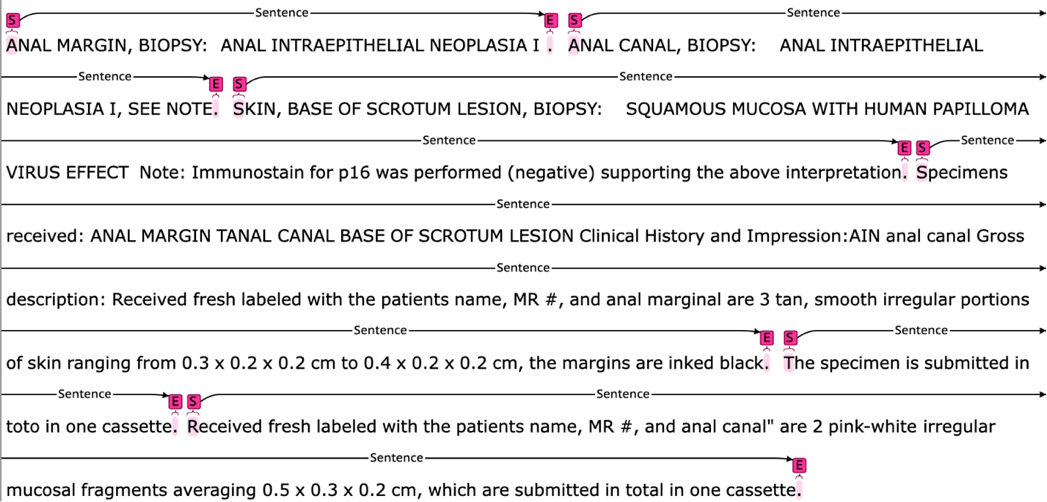


The square "S" marks the start of a sentence, and "E" marks the end. The two annotations are connected with an arrow to highlight each sentence.

**Tokenizer**

The tokenizer component we used in the pipeline is the default tokenizer included in CLAMP (DF_CLAMP_Tokenizer).[2] This tokenizer has been purposely developed for use in medical notes. It identifies and annotates all words and punctuation marks as individual tokens. Figure S2 shows a screenshot with a sample report annotated with the tokenizer.

**Figure S2. Sample biopsy annotated with the tokenizer.**


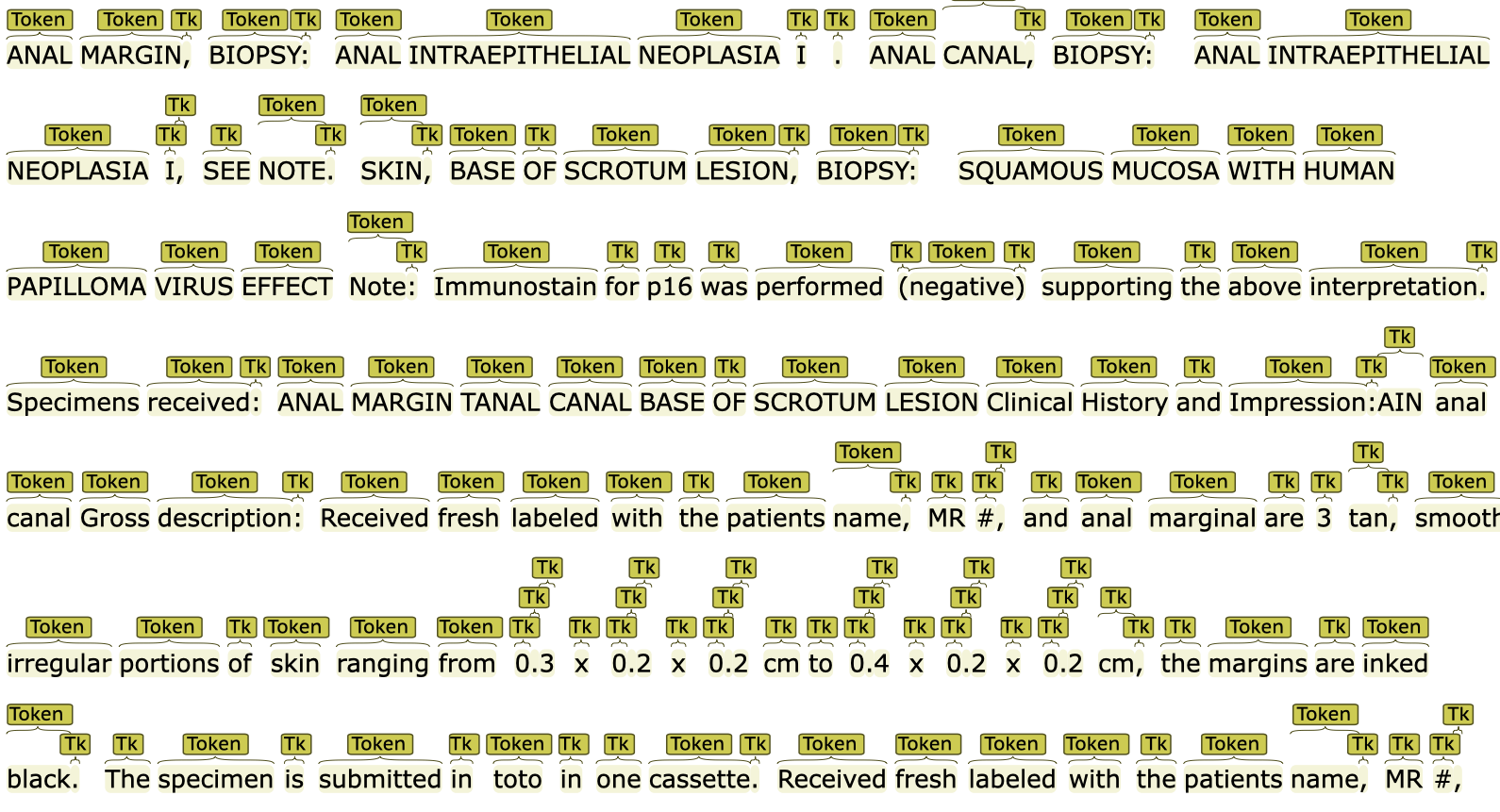


All words and punctuation marks are identified with either a “Token” or “Tk” label.

**Part of Speech Tagger**

The part of speech (POS) tagger assigns a part of speech to each token. The POS tagger component we used in the pipeline is the default is the default POS tagger included with CLAMP. This POS tagger uses a modified version of the OpenNLP POS tagger [3] that has been retrained on clinical notes using machine learning.[4] Figure S3 is a representative screenshot showing an annotation using the POS tagger.

**Figure S3. Representative screenshot showing part of speech tagger**


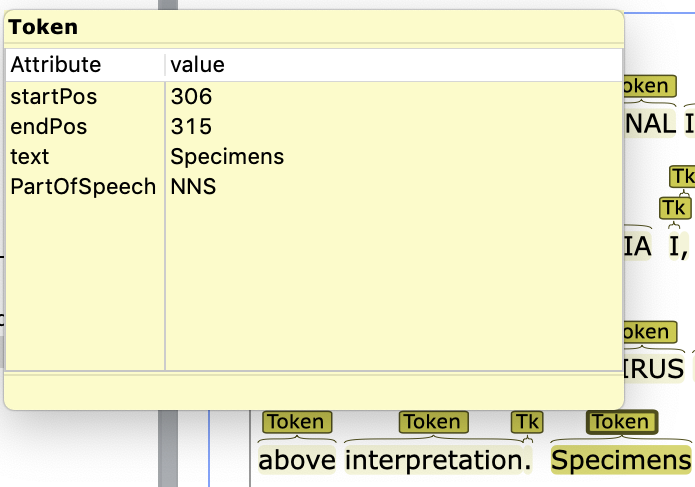


When the token "Specimens" is highlighted, the part of speech (PartOfSpeech) is annotated as “NNS”, which stands for a plural noun.

**Section Identifier**

The section identifier we used in the pipeline is the default section identifier included with CLAMP (DF_Dictionary_based_section_identifier). This is a dictionary-based algorithm that classifies each section into general groups based on section headers. For example, the section header "Procedural Complications" will be assigned to the "Complications" category.

**Assertion and Negation**

To determine whether a tagged expression is being negated or asserted, we used NegEx, an extensively validated negation algorithm.[5] This algorithm uses a dictionary-based method to identify terms denoting negation (e.g., “no sign of,” “no evidence to suggest,” “absence of,”) and reclassifies relevant annotations as either “present” or “absent” based on their proximity to the negation terms. NegEx uses 35 negation phrases to determine whether a term is being asserted or negated.[5] This algorithm is sensitive to double negatives (e.g., “not excluded”), modifiers (e.g., “Dr. Negative”), and vague terminology (e.g., “within normal limits”). Figure S4 is a representative screenshot highlighting a pathologic diagnosis that was annotated using NegEx.

**Figure S4. Representative screenshot showing negation tagger**


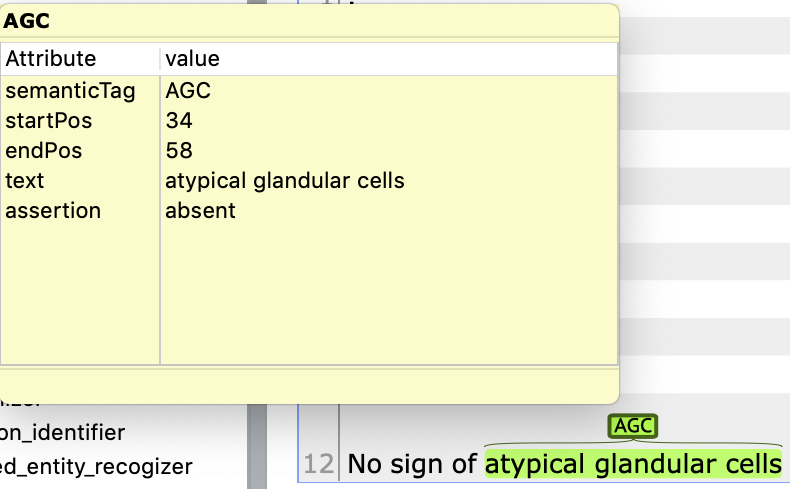


The annotate term in green is atypical glandular cells (AGC), which is classified by the Assertion and Negation algorithm as “absent” because it is preceded by the phrase “no sign of.”

**Named Entity Recognizer**

The named entity recognizer (NER) component we implemented works by finding certain words/phrases in the medical text and annotating them to be further processed. To match concepts in the pathology reports to terms in a dictionary derived from the Unified Medical Language System (UMLS) Metathesaurus (version 2019AA), we first used CLAMP’s default dictionary lookup platform (DF_Dictionary_lookup).[6] While the CLAMP dictionary accounts for multiple variations of a medical words or phrases, it does not account for all variations as they appear on pathology reports. Depending on individual reporting styles, pathologists may assign diagnoses with differing shorthand or preferred terminology. Thus, we enhanced the default NER by adding our own custom HPV dictionary, which included 313 HPV related terms and their associated stems. These terms include possible diagnoses for the report (see Table S1) as well as keywords for determining information about the report (e.g., cytology vs. histology, cervical vs. anal, etc.). This enabled us to account for syntax and vocabulary not otherwise detected with the UMLS dictionary.

**REFERENCES**

1. Kluegl P, Toepfer, M., Beck, P., Fette, G., Puppe, F: **UIMA Ruta: Rapid development of rule-based information extraction applications**. *Natural Language Engineering* 2016, **22**(1):1-40

2. Doan S, Bastarache L, Klimkowski S, Denny JC, Xu H: **Integrating existing natural language processing tools for medication extraction from discharge summaries**. *J Am Med Inform Assoc* 2010, **17**(5):528-531.PMID:20819857

3. Severance C: **The Apache Software Foundation: Brian Behlendorf**. *Computer* 2012, **45**(10):8-9

4. Fan JW, Yang EW, Jiang M, Prasad R, Loomis RM, Zisook DS, Denny JC, Xu H, Huang Y: **Syntactic parsing of clinical text: guideline and corpus development with handling ill-formed sentences**. *J Am Med Inform Assoc* 2013, **20**(6):1168-1177.PMID:23907286

5. Chapman WW, Bridewell W, Hanbury P, Cooper GF, Buchanan BG: **A simple algorithm for identifying negated findings and diseases in discharge summaries**. *J Biomed Inform* 2001, **34**(5):301-310.PMID:12123149

6. Uzuner O, South BR, Shen S, DuVall SL: **2010 i2b2/VA challenge on concepts, assertions, and relations in clinical text**. *J Am Med Inform Assoc* 2011, **18**(5):552-556.PMID:21685143
